# Characterization of Zinc Oxide-Urea Formaldehyde Nano Resin and Its Impact on the Physical Performance of Medium-Density Fiberboard

**DOI:** 10.3390/polym13030371

**Published:** 2021-01-25

**Authors:** Waheed Gul, Syed Riaz Akbar Shah, Afzal Khan, Catalin I. Pruncu

**Affiliations:** 1 Department of Mechanical Engineering, Institute of Space Technology, Islamabad 44000, Pakistan; 2 Department of Mechatronics Engineering, University of Engineering and Technology, Peshawar 25120, Pakistan; rasayed@uetpeshawar.edu.pk; 3 Department of Mechanical Engineering, University of Engineering and Technology, Peshawar 25120, Pakistan; afzalkhan@uetpeshawar.edu.pk; 4 Designs, Manufacturing & Engineering Management, University of Strathclyde, Glasgow G1 1XJ, Scotland, UK; 5 United Kingdom Mechanical Engineering, Imperial College London, Exhibition Rd., London SW7 2AZ, UK

**Keywords:** MDF, physical properties, SEM, XRD, DSC, TGA

## Abstract

The main purpose of this research work is to characterize zinc oxide-urea formaldehyde nano resin and identify the physical performance of medium-density fiberboard (MDF). Considering the dry weight of natural fibers, the ZnO nanoparticles were added to urea formaldehyde (UF) glue at four levels—0.0%, 1.0%, 2.0% and 3.0%—and their effects were investigated in terms of the physical properties of MDF. The surface morphology and crystalline structure of ZnO, UF and UF-ZnO nanofillers were characterized using Scanning Electron Microscopy (SEM) and X-ray diffraction (XRD) analysis and significant improvements were achieved as a result of the addition of nanoparticles. Thermal properties were analyzed by means of differential scanning calorimetry (DSC) and thermogravemetric analysis (TGA) and it was observed that increasing the concentration of ZnO nanoparticles ultimately enhanced the curing of UF-ZnO nanofillers. Finally, density, thickness swelling and water absorption properties were investigated and it was observed that thickness swelling and water absorption properties were improved by 38% and 12%, respectively, when compared to control MDF.

## 1. Introduction

In recent years, extensive scientific research has been conducted on nanoparticles use in furniture industries. Exceptional results had been achieved in the form of physical, mechanical and biological properties of wood-based products due to the large surface area and reactivity of nanoparticles. In most of the research, the researchers used alumina, multi-walled carbon nanotubes (MWCNTs), copper, nanoclay and titanium oxide for dry process board, commercially known as medium-density fiberboard. Medium-density fiberboard is extensively used in the furniture industry due to its smooth and strong surface. However, its physical performance keeps it very limited for external use [1,2]. This work concentrated on ZnO nanoparticles to enhance the physical properties of MDF. It is a universal fact that urea formaldehyde plays a significant role in MDF manufacturing [3]. This resin is mixed with natural fibers in a hot and pressurized environment and behaves as a binder to hold fibers together with enough strength [4].

As hydroxyl groups abundantly present in the cell wall of natural fibers make MDF a weak product for its water absorption and thickness swelling properties [5].MDF used in dry conditions has a low thickness swelling, but there are classes for use in humid conditions, such as Medium Density Fiberboard (MDF). Moreover, in comparison with particleboard, its water resistance is higher. For a 16 mm board, the specification for Thickness swelling TS is 8%, while for Particleboard is 14%. For MDF used in dry conditions, TS is 12% while for PB, it is not specified. It must be added that cross linkages among fibers, lignin cellulose of fibers and poly condensation reactions of urea formaldehyde resin reduced the strength of MDF [6]. However, a flexible cell wall structure allows one to modify natural fibers by means of useful additives [7]. Therefore, several mechanisms were developed in the past to improve its thickness swelling and water absorption properties.

**Figure 1:**
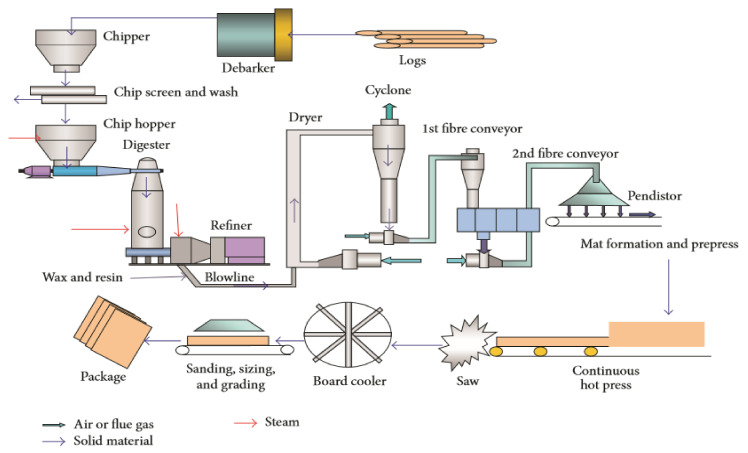
Schematic diagram of MDF Manufacturing Process [2].

Figure 1 shows the manufacturing process of nano MDF (MDF with the adhesive containing nano particles of ZnO) associated with different work stations, i.e., Material preparation, fiber formation, fiber treatment, mat forming, hot pressing, board treatment and warehouse. In the material preparation section, the timber is transformed into chips using chipper machine. The chips are then separated to achieve the vital size. The confirmed chips are then extracted and fed to a chip washer through a drawstring conveyor with an iron remover mounted above it. These chips are formerly splashed to increase their quality. They are then transferred to a fiber parting section. In the fiber parting section, the chips are roasted at a temperature (175–195 °C) at (6–10 bar) pressure for nearly 3–5 min to soften them. Approximately 1–2 (wt %) of paraffin polish is added to the moderated chips to ensure impervious swelling. The moderated chips are then transported to a crushing chamber where the materials are exposed to water and heat, resulting in their disintegration. Pulps are formed and passed through a blow line, where urea formaldehyde resin is introduced into the pulp. After this process, pulp in the form of fiber enters into the dryer. In the fiber curing section, moisture within the obligatory assortment of fibers is evaporated in controlled, indoor conditions. The final moisture content in the fiber is within 8–13 (wt %). In the mat forming section, the fiber is placed uniformly onto the floor-covering belt. Further down the belt, air blowing is applied, and the door mat of a quantified thickness is shaped. The pre-press dispels the air, making it unavailable to the mat and increasing the elastic strength of the mat. In the Board Trimming Unit, the panel is chilled and assigned longitudinal and transversal cuts for cutting. The powder is detached from the edges of the panel. In the Sanding unit, the panel is refined to the essential dimensions by eliminating the superfluous surface. The panel is then examined and moved to a warehouse [2].

Nanoparticles are an example of materials that lead to attractive results, such as a decrease of swelling and lower mass gain when in contact with water [8]. The main aims are to study the physical properties, i.e., water absorption and thickness swelling of MDF, by examining the effects of using different ZnO nanoparticles weight percentages in its resin content. Because of its promising functional properties, ZnO was introduced in this study to create functional flexible surfaces that simultaneously exhibit high water-resistance [9]. A brief literature review of nanoparticle-based composites is illustrated here.

The physical and mechanical performances of nano particleboard were studied by Taghiyari, H. Ret al. (2011) using a silver nano suspension at two different concentration levels in a ratio of 1:1.5 based on the dry weight of fibers. Substantial growth for the 100 and 150 mL/kg suspensions was observed when the hot press time was reduced by 10.9% and 10.1%, respectively [10].

Another study was conducted by Xian, D et al. (2013) in which he used 2% nanoclay in melamine formaldehyde (MF) resin for particleboard production. A 6% improvement in the thickness swelling property was observed in this research [11]. Nanowollastonite was used by Taghiyari, H. R et al. (2014) as nanofillers with urea formaldehyde. The thermal conductivity of the final nano MDF was enhanced due to fast curing of urea formaldehyde resin [12]. Nano MDF containing three nanoparticles, namely zinc oxide, alumina and silicon dioxide, was used by Candan, Z et al. (2015). It was observed that addition with 1% of these nanoparticles resulted in an enhanced elastic and rupture moduli [13].

Taghiyari et.al. (2016) used wollostonite and camel fibers in 30 and 10 wt % ratios respectively with other natural fibers. Enhanced physical and mechanical properties were obtained in terms of water absorption, bending strength and internal bonding [14]. Physical and mechanical performances of particleboard were investigated by Ismita et al. (2017) by adding nanoclay as a nanofiller in urea formaldehyde resin [15]. Yipeng Chen et al. (2018) used calcium carbonate and plastic as additives in urea formaldehyde resin and highlighted improvements in water absorption and bending strength compared to control board [16]. Alabduljabbar, H et al. (2020) determined the physical and mechanical properties of nano MDF with added alumina nanoparticles. A considerable improvement was reported in the physical and mechanical properties at a concentration of 4.5% alumina nanoparticles in urea formaldehyde resin [17].

Furthermore, in a comprehensive literature review, it has been observed that the physical properties of MDF were not significantly improved. A novel idea to improve the physical properties of MDF by the addition of ZnO nanoparticles in urea formaldehyde resin is presented in this work.

## 2. Materials and Methods

### 2.1. Materials

The raw materials for nano MDF are Populus caspica fibers, zinc oxide nanoparticles and urea formaldehyde resin. Populus caspica fibers were obtained from Ciel Woodowrks pvt. Ltd., Peshawar, Pakistan. Zinc oxide nanoparticles in white powder form (99.80% pure) were supplied by CCL Minal and Chemical Company Jawa Timur, Indonesia. The average particle size of ZnO was 95 nm. Urea formaldehyde resin was purchased from WAH Noble Chemical Company, Wah Cantt, Taxila, Pakistan.

### 2.2. Urea Formaldehyde and Zinc Oxide (UF-ZnO) Nanofiller Preparation

Urea formaldehyde and zinc oxide (UF-ZnO) nanofillers were produced in the Materials Science Lab, Energy Centre, UET, Peshawar, Pakistan. Table 1 illustrates the composition of these nanofillers.

A known weight of 200 g of urea formaldehyde resin was mixed with 0%, 1%, 2% and 3% wt ZnO nanoparticles. For stirring and uniform mixing, sonication was applied for 35 min. No agglomeration was observed in the final solution. The samples were named ZnO_0_, ZnO_1_, ZnO_2_, and ZnO_3_ due to the concentration levels of ZnO.

### 2.3. Design of Nanocomposite Containing (UF-ZnO) Nanofiller

Populus caspica fibers were mixed with resin and ZnO nanoparticles at various concentrations and manufactured in the laboratory. In the preparation of the pre-press mat, resin weighing 10% of the weight of the wood fibers was mixed with tropical hardwood fibers with the aid of a spray gun in a rotary drum. The platens subjected the mat to hot pressing at 175 °C at a pressure of 165 bar for 4.1 min. Panels measuring 450 × 450 × 18 mm^3^ manufactured in this manner had an average density of 630–640 kg/m^3^. The panels were then conditioned to a relative humidity of 65 ± 5% and a temperature of 20 °C to attain uniform moisture content in the panels. The panels were trimmed to determine the thickness swelling and water absorption and to estimate the density.

## 3. Characterization

Urea formaldehyde and zinc oxide (UF-ZnO) nanofillers were sonicated with the help of a Ultrasonic Processor UP 400S of Hielscher Ultrasound Technology Company, coulmbus, USA for 35 min. Scanning Electron Microscopy (SEM) of ZnO nanoparticles and UF-ZnO nanofillers was conducted using MIRA3 (TESCAN, Brno, Czech Republic) with a testing voltage of 15 kV and a 55 s counting time. A gold sputtering technique was applied using a Safematic CCU-010 Gold/Carbon Sputter, (Labtech International Ltd., and Heathfield, UK) before SEM was conducted. X-ray diffraction (XRD) of ZnO nanoparticles and UF-ZnO nanofillers was achieved using a GNR X-Ray Explorer, Analytical Instrument Group, Novara, Italy with an output power of 3 kW, output voltage of 60 kV, output current of 60 mA and 2θ range from 10–80°. Fourier Transform Infrared Spectroscopy (FTIR) of UF resin and UF-ZnO nanofillers was carried out using Shimadzu IR Prestige-21Analytical and Measuring Instruments, North America with a 30° incident angle equipped with a Germanium-coated KBr plate, resolution of 16 cm^−1^ and wave number range of 500–3500 cm^−1^.Thermogravmetric analysis (TGA) and Differential scanning calorimetry (DSC) were obtained using a Mettler Toledo TGA/DSC-1-star apparatus, Polaris Parkway, Columbus, USA with a temperature range of 0–700 °C (for TGA) and 0–1600 °C (for DSC) with a heating rate of 10.0 °C/min in a Nitrogen tributary of 10.5 mL/min. The physical properties, i.e., density, thickness in swelling and water absorption, of nano MDF sheets were investigated according to EN, B. 323, (1993) [18], EN 317, (1993) [19] and ASTM D570-98 (2010) [20] respectively. The Tukey method with 95% confidence was applied for Analysis of Variance (ANOVA) with the help of origin 9.0, 64-bit software of Origin Lab Corporation Northampton, Northampton, MA, USA.

## 4. Results and Discussion

### 4.1. Scanning Electron Microscopy (SEM) of ZnO Nanoparticles

**Figure 2:**
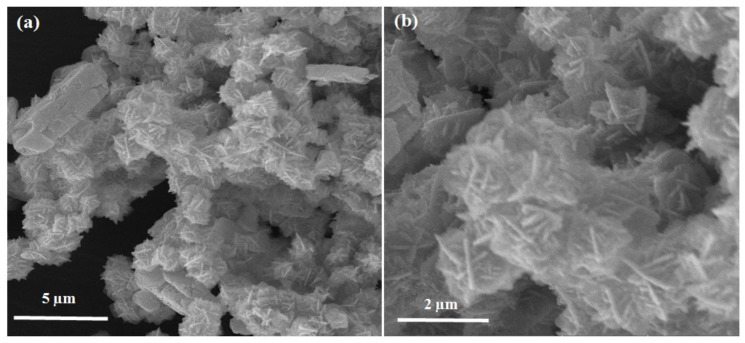
Scanning electron microscopy of zinc oxide nanoparticles at (**a**) 10,000×, (**b**) 25,000× magnifications.

Figure 2 shows the Scanning Electron Microscope (SEM) images of the zinc oxide nanoparticles at magnifications of (a) 10,000×, (b) 25,000×. It has been observed that the particles are uniform, flower-like shapes and very few of them have a cubic morphology. It is clear from Figure 2 that the flower-like shapes arise via a few different routes and are therefore predominant, as reported by Saikia, L. et al. 2014 [21]. Every region contains a small number of nanorods with an approximately consistent diameter and resemble pencils in terms of their shape.

### 4.2. X-ray Diffraction Analysis of ZnO Nanoaprticles

**Figure 3:**
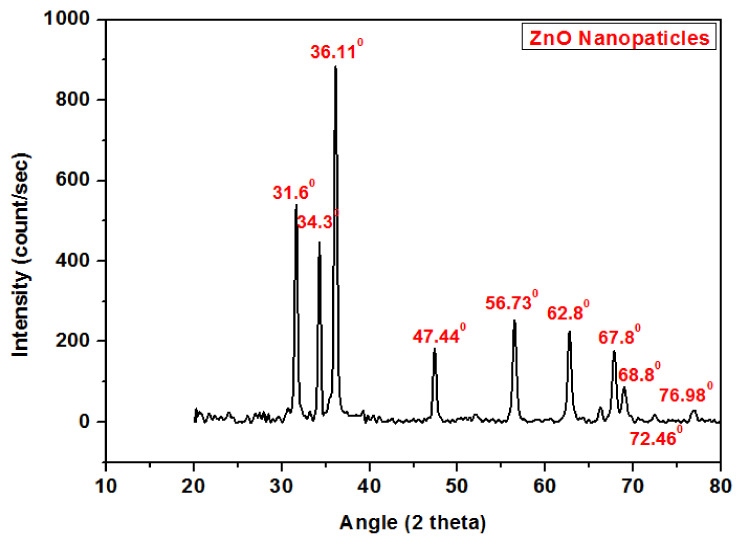
X-ray diffraction analysis of zinc oxide nanoparticles.

Figure 3 shows the X-Ray Diffraction (XRD) analysis of zinc oxide (ZnO) nanoparticles. It was observed that ZnO nanoparticles had corresponding diffraction peaks at 31.6°, 34.3°, 36.11°, 47.44°, 56.73°, 62.8°, 67.8°, 68.8°, 46.72°and 76.98° as reported by Ramimoghadam, D et al. (2013) [22], Lepot, N et al. (2007) [23] and H. Alshamsi et al. (2018) [24]. The peak at 2θ = 36.11° is the most intense and can be compared with the peaks at 31.6° and 34.3°. The peaks at 2θ = 47.44°, 56.73° and 62.8° are almost identical, with low intensities. The peaks at 31.6°, 34.3°, 36.11°, 47.44°, 56.73°, 62.8°, 67.8°, 68.8° 72.46° and 76.98° correspond to lattice planes (100), (002), (101), (102), (110), (103), (200), (112), (004) and (002).

### 4.3. Scanning Electron Microscopy of Cured UF-ZnO Nanofiller

**Figure 4:**
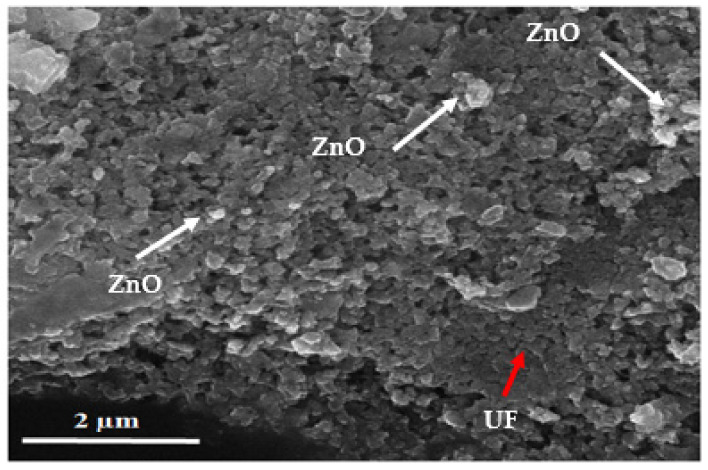
Scanning electron microscopy image of cured UF-ZnO nanofillers.

Figure 4 shows the scanning electron microscopy image of cured UF-ZnO nanofiller. Apeculi arcon figuration of linkages of the urea formaldehyde glue was noticed and perceptible partial ditches were investigated. These ditches were covered by zinc oxide nanoparticles in the resin. The nano MDF becomes high-strength as a result of the filling of superfluous pores and gaps by zinc oxide nanoparticles, as reported by Gul, W. et al. (2020) [25]. The white visible region in the scanning electron microscopy image confirmed the presence of zinc oxide nanoparticles and the black region indicates urea formaldehyde glue.

### 4.4. X-ray Diffraction Analysis of UF-ZnO Nanofiller

**Figure 5:**
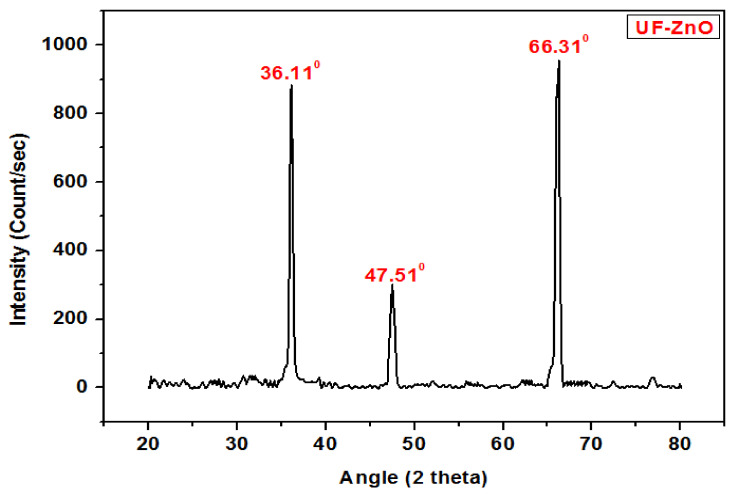
X-ray diffraction analysis of UF-ZnO nanofillers.

The X-Ray diffraction pattern of UF-ZnO nanofillers shown in Figure 5 indicates the crystalline nature of the sample. The peaks at 36.11° and 66.31°, corresponding to lattice planes (101) and (200) of ZnO, are present in the diffraction pattern of the cured UF-ZnO nanofillers but at a slightly lower intensity. This is because UF resin is amphorous in nature and hence reduces the crystallinity of ZnO. This observation provides evidence that UF-ZnO nanofillers were formed.

### 4.5. Fourier-Transform Infrared (FT-IR) Spectroscopy of UF and UF-ZnO Nanofillers

**Figure 6:**
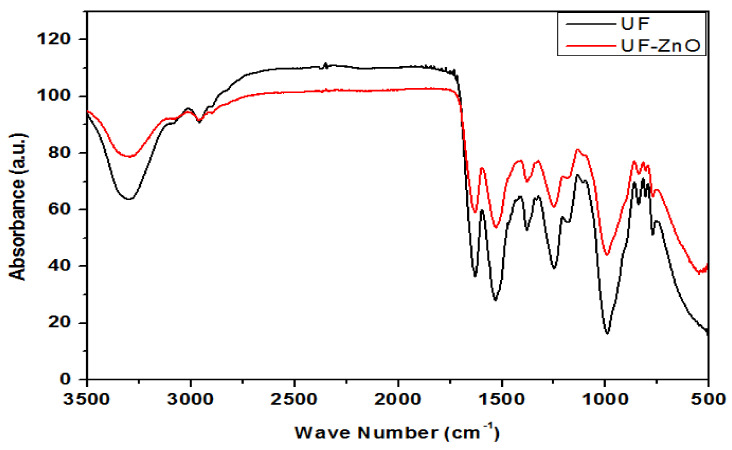
Fourier-transform infrared (FT-IR) spectroscopy of ZnO and UF-ZnO nanofillers.

Figure 6 shows the FT-IR spectra of urea formaldehyde resin and UF-ZnO nanofillers.

Specific bands along with their respective functional groups are summarized in Table 2. The FT-IR spectrum of urea formaldehyde contains bands at 3312, 2960, 1619, 1521, 1248, 990, 843 and 763 cm^−1^ which correspond to NH, CH, CO and OH groups in the array 3400–3300, 3000–2900 cm^−1^, 1700–1600 cm^−1^ and 1550–1500 cm^−1^. The wave number for CH_2_OH, CH_3_ and CN groups is the range 1300 cm^−1^ to 1250 cm^−1^. The shift in the band position of NH, CH and CO in the sample with UF towards lower wavelengths indicates that ZnO weakly interacts with UF at NH sites, because ZnO acts as a Lewis acid, while NH acts as a Lewis base. This acid–base interaction ultimately increases the density in the cross-linkage configuration and provides stability to zinc oxide nanoparticles in urea formaldehyde resin, as reported by Ozdemir, F. A. et al. (2009) [26], Sun, X et al. (2002) [27] and pandey, N. et al. (2015) [28].

### 4.6. Differential Scan Calorimetry (*DSC*) of UF-ZnO Nanofillers

**Figure 7:**
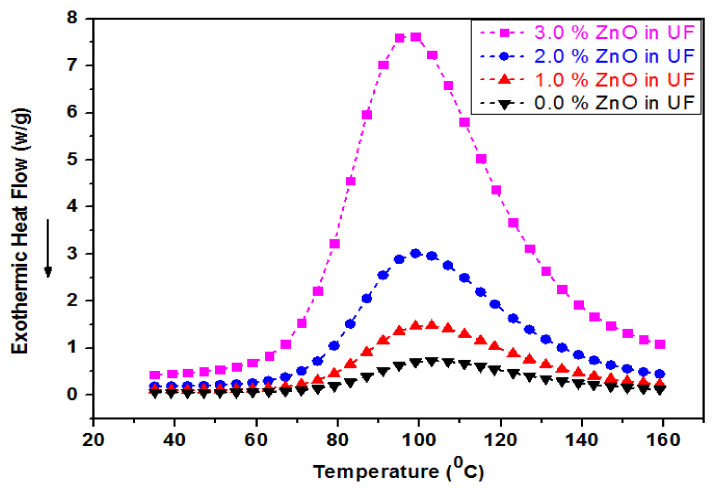
Differential Scan Calorimetry (*DSC*) of UF-ZnO Nanofillers.

Figure 7 shows the differential scan calorimetry (*DSC*) of UF-ZnO nanofillers with 0.0%, 1.0%, 2.0% and 3.0% concentrations of ZnO nanoparticles.

The relationship between temperature and exothermic heat flow for all samples can be seen in the DSC curves. An inverse relationship between therapeutic temperature and zinc oxide nanoparticle absorption was experimentally investigated. As the absorption of ZnO nanoparticles enhances, the aeration temperature decreases and at the same time the total heat content increases with ZnO nanoparticle concentration in a directly proportional manner. The peak at 99 °C for a concentration of 3.0% ZnO nanoparticles was achieved due to supplementary bonding produced in the UF resin, which raised the heat transfer and curing rate. The increase in the intensity of the endothermic peak at 99 °C can result either from an increase in water release (condensation reactions) or lower heat release from the reaction, because the peak is the result of the addition of two phenomena: exothermic reaction and endothermic water vaporization. The same result had previously been reported for the same resin by Gul, W. et al. (2020) [29].

### 4.7. Thermogravmetric Analysis (TGA)Analysis of of UF-ZnO Nanofillers

**Figure 8:**
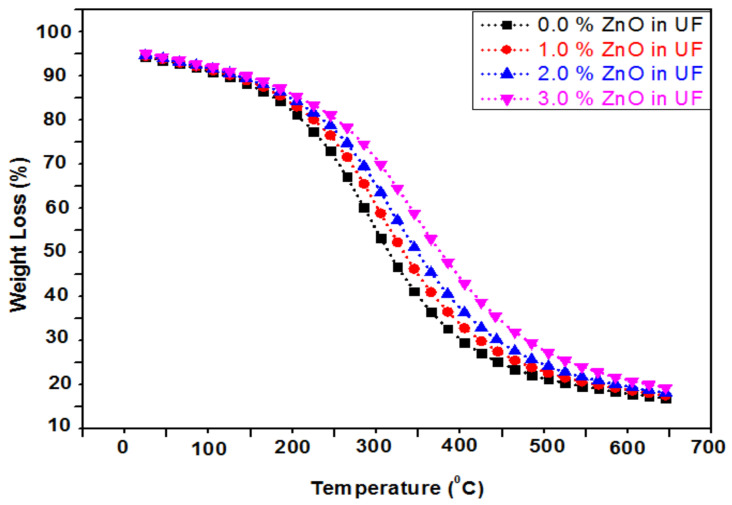
Thermogravimetric analysis (TGA) analysis of UF-ZnO nanofillers.

Figure 8 shows thermogravimetric analysis (TGA) of UF-ZnO nanofillers with 0.0%, 1.0%, 2.0% and 3.0% concentrations of ZnO nanoparticles. The relationship between temperature and % weight loss was analyzed through the TGA curves. As the temperature increased in the range of 25−160 °C, vaporization occurred and a small weight loss was observed due to resin curing, as reported by Alabduljabbar et al. (2020) [17]. This is because of inter- and intramolecular interactions that result in the formation of carbon and hydrogen bonds.

Upon the addition of ZnO nanoparticles, a strong internal bonding and van der Waals forces formed among the functional groups of concentrated resin, leading to a high thermal stability. The degradation happens within a temperature range of 160 °C to 500 °C. The concentrated resin was hydrolyzed and its intermolecular interface toughened, which ultimately increased the peak zone. From the TGA results, it is notable that there was a decrease in weight loss with increasing ZnO concentration. A smaller weight loss corresponded to a lower degree of condensation (less water produced).

### 4.8. Analysis of Variance (ANOVA) of Nano-MDF for Physical Properties

A one-way ANOVA for the density of nano MDF for 0.0%, 1.0% 2.0% and 3.0% concentrations of ZnO nanoparticles is summarized in Table 3. Nano MDF with 0.0% ZnO had a mean density of 637 kg/m^3^, holding a variance of 57. The average densities were 643, 635 and 639 kg/m^3^ for 1.0%, 2.0% and 3.0% with variances of 72, 165, and 114, respectively. The reported density values were different and the one-way ANOVA described the probability (*p*-value) reached 0.78.

Table 4 describes one-way ANOVA of the thickness swelling property for 0.0%, 1.0% 2.0% and 3.0% concentrations of ZnO nanoparticles. Variance of 2.33 and thickness swelling of 25.33% were recorded for ZnO (0.0%). Similarly, the thickness swelling average values for 1.0% 2.0% and 3.0% concentrations of ZnO nanoparticles were observed to be 22.26%, 17.66% and 15.63% with variances of 0.41, 1.42 and 1.40, respectively, along with 3.18 × 10^−5^ (*p*-value).

The one-way statistical analysis of water absorption is reported in Table 5. Three iterations of this property were tested for 0.0%, 1.0%, 2.0%, and 3.0% ZnO nanoparticle concentrations. An average value of 44.66% with a variance of 17.33 was observed for MDF without ZnO nanoparticle concentration.

Average values of 44%, 39.89% and 29.3% for the thickness swelling with a variance of 9, 7.69 and 34.77 were noted for nano MDF with 1.0% 2.0% and 3.0% concentrations of ZnO nanoparticles. These thickness swelling values changed for different concentrations and the one-way factor ANOVA indicates the probability (*p*-value 0.006).

### 4.9. Final Average Physical Properties of UF-ZnO-Based MDF

Nano MDF was manufactured with 0.0%, 1.0%, 2.0%, and 3.0% ZnO nanoparticles in UF resin. Each specimen was experimentally tested for density thickness swelling and water absorption properties with three iterations and the mean values are tabulated in Table 6. The thickness swelling and water absorption properties were investigated for 24 h following the British Standards EN-3171993 and ASTM D517, respectively.

The density rise and fall as a result of the increased inclusion of nanofillers was due to nanofiller propagation. A secure decrease in the thickness swelling values of the specimen for 24 h was recorded. This is owing to the reduction of pores in the nano MDF boards. The water absorption property also decreased with increasing ZnO concentration due to mature drying of the specimen in the hot press.

## 5. Conclusions

The characterization of zinc oxide-urea formaldehyde nano resin and its impact on the physical performance of medium-density fiberboard were experimentally investigated. The results indicate that the addition of zinc oxide nanoparticles enhanced the thickness swelling and water absorption properties of nano MDF. It is concluded that well-penetrated ZnO nanoparticles contained within urea formaldehyde enhanced the pores among the resin matrix and nano MDF, improving the thickness swelling and water absorption properties. Because of the promising functional properties, ZnO was introduced in this study to create functional flexible surfaces that simultaneously exhibit high water-resistance. It can also be stated that fast curing and heat transfer of the resin results in high production.

This research can be further enhanced in future by adding other nanoparticles, i.e., graphene to the UF-ZnO nanofillers.

## Figures and Tables

**Table 1:** Urea formaldehyde and zinc oxide (UF-ZnO) nanofiller compositions.

	Composition			
Materials	ZnO_0_	ZnO_1_	ZnO_2_	ZnO_3_
UF (gram)	200	200	200	200
ZnO (wt %)	0	1	2	3

**Table 2:** Summary of Fourier-transform infrared (FT-IR) spectroscopy of ZnO and UF-ZnO Nanofillers.

UF	UF-ZnO	Stretching/Bending Vibrations
3312	3308	(–NH_2_) group
2960	2957.7	Stretching vibrations for C–H
1619	1627	Stretching vibrations of C=O bonds
1521	1529	stretching vibrations of –OH
990	993	–CO bending
843	840	Bending vibrations for C–H bond

**Table 3:** Density values of Nano MDF containing UF-ZnO nanofillers.

	Groups	Iteration	Sum	Average	Variance	
	ZnO (0.0%)	3	1911	637	57	
	ZnO (1.0%)	3	1930	643.33	72.33	
	ZnO (2.0%)	3	1906	635.33	165.33	
	ZnO (3.0%)	3	1918	639.33	114.33	
ANOVA						
Source of Variation	SS	df	MS	F	*p*-value	F crit
Between Groups	108.25	3	36.08	0.35	0.78	4.06
Within Groups	818	8	102.25			
Total	926.25	11				

**Table 4:** Thickness swelling values of nano MDF containing UF-ZnO nanofillers.

	Groups	Iteration	Sum	Average	Variance	
	ZnO (0.0%)	3	76	25.33	2.33	
	ZnO (1.0%)	3	66.8	22.26	0.41	
	ZnO (2.0%)	3	53	17.66	1.42	
	ZnO (3.0%)	3	46.9	15.63	1.40	
ANOVA						
Source of Variation	SS	df	MS	F	*p*-value	F crit
Between Groups	173.67	3	57.89	41.54	3.18 × 10^−5^	4.06
Within Groups	11.14	8	1.39			
Total	184.82	11				

**Table 5:** Water Absorption values of ZnO-UF MDF for different iterations.

	Groups	Iteration	Sum	Average	Variance	
	ZnO(0.0%)	3	134	44.66	17.33	
	ZnO (1.0%)	3	132	44	9	
	ZnO (2.0%)	3	119.67	39.89	7.69	
	ZnO (3.0%)	3	87.9	29.3	34.77	
ANOVA						
Source of Variation	SS	df	MS	F	p-value	F crit
Between Groups	453.39	3	151.13	8.78	0.006	4.06
Within Groups	137.59	8	17.19			
Total	590.99	11				

**Table 6:** Final physical properties of UF-ZnO-based MDF.

MDF Specimen	Density (kg/m^3^)	TS *	WA *
S_0.0_ZnO_0.0_	637	25.33	44.66
S_1.0_ZnO_1.0_	643	22.26	44
S_2.0_ZnO_2.0_	635	17.66	39.89
S_3.0_ZnO_3.0_	639	15.63	39.3
Standard	720 ± 20	≤12	<45

* 24, density (EN-323 standard) [18], TS (EN-317 standard) [19], WA (ASTM D570 standard) [20].
